# Formation of Foamy Macrophages by Tuberculous Pleural Effusions Is Triggered by the Interleukin-10/Signal Transducer and Activator of Transcription 3 Axis through ACAT Upregulation

**DOI:** 10.3389/fimmu.2018.00459

**Published:** 2018-03-09

**Authors:** Melanie Genoula, José Luis Marín Franco, Maeva Dupont, Denise Kviatcovsky, Ayelén Milillo, Pablo Schierloh, Eduardo Jose Moraña, Susana Poggi, Domingo Palmero, Dulce Mata-Espinosa, Erika González-Domínguez, Juan Carlos León Contreras, Paula Barrionuevo, Bárbara Rearte, Marlina Olyissa Córdoba Moreno, Adriana Fontanals, Agostina Crotta Asis, Gabriela Gago, Céline Cougoule, Olivier Neyrolles, Isabelle Maridonneau-Parini, Carmen Sánchez-Torres, Rogelio Hernández-Pando, Christel Vérollet, Geanncarlo Lugo-Villarino, María del Carmen Sasiain, Luciana Balboa

**Affiliations:** ^1^Laboratorio de Inmunología de Enfermedades Respiratorias, Instituto de Medicina Experimental (IMEX)-CONICET, Academia Nacional de Medicina, Buenos Aires, Argentina; ^2^International Associated Laboratory (LIA) CNRS IM-TB/HIV (1167), Toulouse, France; ^3^International Associated Laboratory (LIA) CNRS IM-TB/HIV (1167), Buenos Aires, Argentina; ^4^Institut de Pharmacologie et de Biologie Structurale, Université de Toulouse, CNRS, UPS, Toulouse, France; ^5^Laboratorio de Fisiología de los Procesos Inflamatorios, Instituto de Medicina Experimental (IMEX)-CONICET, Academia Nacional de Medicina, Buenos Aires, Argentina; ^6^Instituto Prof. Dr. Raúl Vaccarezza, Hospital de Infecciosas Dr. F. J. Muñiz, Buenos Aires, Argentina; ^7^Sección de Patología Experimental, Departamento de Patología, Instituto Nacional de Ciencias Médicas y Nutrición Salvador Zubirán, Mexico City, Mexico; ^8^Departamento de Biomedicina Molecular, Centro de Investigación y de Estudios Avanzados del Instituto Politécnico Nacional, Mexico City, Mexico; ^9^Fundación Instituto Leloir, CABA, Buenos Aires, Argentina; ^10^Laboratory of Physiology and Genetics of Actinomycetes, Instituto de Biología Molecular y Celular de Rosario (IBR-CONICET), Facultad de Ciencias Bioquímicas y Farmacéuticas, Universidad Nacional de Rosario, Rosario, Argentina

**Keywords:** ACAT, interleukin-10, foamy macrophages, lipids, signal transducer and activator of transcription 3, tuberculosis

## Abstract

The ability of *Mycobacterium tuberculosis* (Mtb) to persist in its human host relies on numerous immune evasion strategies, such as the deregulation of the lipid metabolism leading to the formation of foamy macrophages (FM). Yet, the specific host factors leading to the foamy phenotype of Mtb-infected macrophages remain unknown. Herein, we aimed to address whether host cytokines contribute to FM formation in the context of Mtb infection. Our approach is based on the use of an acellular fraction of tuberculous pleural effusions (TB-PE) as a physiological source of local factors released during Mtb infection. We found that TB-PE induced FM differentiation as observed by the increase in lipid bodies, intracellular cholesterol, and expression of the scavenger receptor CD36, as well as the enzyme acyl CoA:cholesterol acyl transferase (ACAT). Importantly, interleukin-10 (IL-10) depletion from TB-PE prevented the augmentation of all these parameters. Moreover, we observed a positive correlation between the levels of IL-10 and the number of lipid-laden CD14^+^ cells among the pleural cells in TB patients, demonstrating that FM differentiation occurs within the pleural environment. Downstream of IL-10 signaling, we noticed that the transcription factor signal transducer and activator of transcription 3 was activated by TB-PE, and its chemical inhibition prevented the accumulation of lipid bodies and ACAT expression in macrophages. In terms of the host immune response, TB-PE-treated macrophages displayed immunosuppressive properties and bore higher bacillary loads. Finally, we confirmed our results using bone marrow-derived macrophage from IL-10^−/−^ mice demonstrating that IL-10 deficiency partially prevented foamy phenotype induction after Mtb lipids exposure. In conclusion, our results evidence a role of IL-10 in promoting the differentiation of FM in the context of Mtb infection, contributing to our understanding of how alterations of the host metabolic factors may favor pathogen persistence.

## Introduction

Tuberculosis (TB) is a highly contagious disease caused by *Mycobacterium tuberculosis* (Mtb) infection. Even though the treatment of the disease has been standardized for a while, TB still remains one of the top 10 causes of death worldwide with 10.4 million new cases and 1.3 million deaths from TB among HIV-negative people in 2016 (1). Chronic host–pathogen interaction in TB leads to extensive metabolic remodeling in both the host and the pathogen (2). In fact, the success of Mtb as a pathogen derives from its efficient adaptation to the intracellular milieu of human macrophages. An important strategy to reach this metabolic adaptation is the promotion of lipid body accumulation by the host macrophage leading to foamy macrophages (FM) differentiation. The formation of lipid-laden macrophages is caused by infectious agents through deregulation in the balance between the influx and efflux of lipids. Key for the biogenesis of lipid bodies is the enzyme acyl CoA:cholesterol acyltransferase (ACAT), which represents an ideal target for pathogens (3).

Lipid body accumulation within leukocytes is a common feature in both clinical and experimental infections, especially in mycobacterial infections (4, 5). Mtb infection leads to the induction of FM, a process which is promoted by several mycobacterial lipids (6–8). This event enables the fusion between Mtb-containing phagosomes and lipid bodies resulting in an abundant supply of lipids for the pathogen (7), allowing Mtb to switch into a dormancy phenotype and to become tolerant to several front-line antibiotics (9). For this reason, lipid bodies are considered to be a secure niche for Mtb conferring protection from bactericidal mechanisms, such as respiratory burst (10). Moreover, the presence of FM within granulomatous structures was demonstrated in both experimentally infected animals and patients, especially in individuals developing secondary TB (5, 11). Therefore, FM may play a central role in mycobacterial persistence and reactivation (12, 13).

Concerning the impact of FM on the host immunity against Mtb, it was shown that human macrophages exposed to lipids prior to Mtb infection failed to produce TNF-α and to clear the infection (14, 15). Taking into account that FM generated prior to Mtb infection impair the host immune response, there is a keen interest to identify the host-derived cytokines released at the site of Mtb infection, and to understand how these signals contribute to FM differentiation and alter host defense against Mtb. In this regard, it is well known that different activation programs in macrophages driven by pro or anti-inflammatory cytokines are associated to changes in the lipid metabolism (16). Therefore, it is likely that host cytokines produced in response to Mtb infection contribute to lipids turnover promoting FM formation, and consequently lead to Mtb persistence.

In this work, we report that a TB-associated microenvironment induces FM differentiation program dependent partially on the interleukin-10 (IL-10)/signal transducer and activator of transcription 3 (STAT3) axis through ACAT upregulation. Our approach was to model a genuine TB-associated microenvironment by employing a physiological relevant sample derived from active TB patients, such as the acellular fraction of tuberculous pleural effusions (TB-PE). Indeed, TB-PE are manifested in up to 30% of patients with TB, and they are caused by the spread of Mtb into the pleural space and subsequent local inflammation and recruitment of leukocytes (17). Based on this, we show that the acquisition of the foamy phenotype with immunosuppressive properties involves high IL-10 release, low TNF-α production, impaired Th1 activation, and high bacillary loads. We also confirmed that IL-10-deficiency in bone marrow-derived macrophages (BMDM) prevented partially foamy phenotype induction upon exposure to Mtb lipids. In conclusion, our results provide evidence for a role of IL-10 in promoting foamy differentiation of macrophages in the context of Mtb infection.

## Materials and Methods

### Bacterial Strain and Antigens

*Mycobacterium tuberculosis* H37Rv strain was grown at 37°C in Middlebrook 7H9 medium (Difco) supplemented with 10% albumin-dextrose-catalase (Difco) and 0.05% Tween-80 (Sigma-Aldrich). The Mtb γ-irradiated H37Rv strain (NR-49098) and its total lipids’ preparation (NR-14837) were obtained from BEI Resource, USA.

### Preparation of Human Monocyte-Derived Macrophages (MDM)

Buffy coats from healthy donors were prepared at *Centro Regional de Hemoterapia Garrahan* (Buenos Aires, Argentina) according to institutional guidelines (resolution number CEIANM-664/07). Informed consent was obtained from each donor before blood collection. Peripheral blood mononuclear cells were obtained by Ficoll gradient separation on Ficoll-Paque (GE Healthcare). Then, monocytes were purified by centrifugation on a discontinuous Percoll gradient (Amersham) as previously described (18). After that, monocytes were allowed to adhere to 24-well plates (Costar) at 5 × 10^5^ cells/well for 1 h at 37°C in warm RPMI-1640 medium (GIBCO). The cells were then washed with warm PBS twice. The final purity was checked by fluorescence-activated cell sorting analysis using an anti-CD14 monoclonal antibody (mAb) and was found to be >90%. The medium was then supplemented to a final concentration of 10% fetal bovine serum (FBS, Sigma-Aldrich) and human recombinant Macrophage Colony-Stimulating Factor (M-CSF, Peprotech) at 10 ng/ml. Cells were allowed to differentiate for 5–7 days.

### Preparation of Pleural Effusion (PE) Pools

Pleural effusions were obtained by therapeutic thoracentesis by physicians at the *Hospital F. J Muñiz* (Buenos Aires, Argentina). The research was carried out in accordance with the Declaration of Helsinki (2013) of the World Medical Association, and was approved by the Ethics Committees of the *Hospital F. J Muñiz* and the *Academia Nacional de Medicina de Buenos Aires* (protocol number: NIN-1671-12). Written informed consent was obtained before sample collection. We collected a total of 43 PE samples which were classified according to their etiology, being 38 of them associated to TB (TB-PE) and 5 to heart failure (HF-PE). Individual samples of confirmed TB patients were used for correlation analysis. A group of samples (*n* = 10) were pooled and used for *in vitro* assays to treat macrophages. The selection of these samples was based merely on practical reasons, involving those samples that have been collected earlier through the course of this study. The diagnosis of TB pleurisy was based on a positive Ziehl–Nielsen stain or Lowestein–Jensen culture from PE and/or histopathology of pleural biopsy, and was further confirmed by an Mtb-induced IFN-γ response and an ADA-positive test (19). Exclusion criteria included a positive HIV test, and the presence of concurrent infectious diseases or non-infectious conditions (cancer, diabetes, or steroid therapy). None of the patients had multidrug-resistant TB. Those PE samples derived from patients with pleural transudates secondary to heart failure (HF-PE, *n* = 5) were employed to prepare a second pool of PE, used as control of non-infectious inflammatory PE. The PE were collected in heparin tubes and centrifuged at 300 *g* for 10 min at room temperature without brake. The cell-free supernatant was transferred into new plastic tubes, further centrifuged at 12,000 *g* for 10 min and aliquots were stored at −80°C. After having the diagnosis of the PE, pools were prepared by mixing same amounts of individual PE associated to a specific etiology. The pools were decomplemented at 56°C for 30 min, and filtered by 0.22 µm in order to remove any remaining debris or residual bacteria.

### FM Induction

Macrophages were plated on glass coverslips within a 24-well tissue culture plate (Costar) at a density of 5 × 10^5^ cells/ml per well with or without 20% v/v of PE, 10 µg/ml of Mtb lipids (BEI resources) or infected with Mtb (MOI 2:1) for 24 h. When indicated, cells were pre-incubated with either Cucurbitacin I (50–100 nM, Sigma-Aldrich), or the STAT3 inhibitor Stattic (1–20 µM, Sigma-Aldrich), or the ACAT inhibitor Sandoz 58-035 (5–50 µg/ml, Sigma-Aldrich) for 2 h prior TB-PE addition and for further 24 h during TB-PE incubation. DMSO alone was used as control. Alternatively, cells were treated with recombinant human IL-10 (Peprotech) at the indicated doses. Foam cell formation was followed by Oil Red O (ORO) staining (Sigma-Aldrich) as previously described (20) at 37°C for 1–5 min, and washed with water three times. For the visualization of the lipid bodies, slides were prepared using the aqueous mounting medium Poly-Mount (Polysciences), observed *via* light microscope (Leica) and finally photographed using the Leica Application Suite software. Alternatively, after fixation, cells were labeled with 1 µg/ml of BODIPY 493/503 (Life technologies) for 15 min in order to visualize the lipid bodies by green fluorescence emission using a confocal microscope (Olympus BX51).

### Infection of Human Macrophages with Mtb

Infections were performed in the biosafety level 3 laboratory at the *Unidad Operativa Centro de Contención Biológica, ANLIS-MALBRAN* (Buenos Aires), according to the biosafety institutional guidelines. Macrophages seeded on glass coverslips within a 24-well tissue culture plate (Costar) at a density of 5 × 10^5^ cells/ml were infected with Mtb H37Rv strain at a MOI of 2:1 during 1 h at 37°C. Then, extracellular bacteria were removed gently by washing with pre-warmed PBS, and cells were cultured in RPMI-1640 medium supplemented with 10% FBS and gentamicin (50 µg/ml) for 24 h. The glass coverslips were fixed with PFA 4% and stained with ORO, as was previously described.

### Quantification of Total Cholesterol

Total cholesterol was determined in TB-PE or cell lysates using the *Colestat Enzimatico kit* according to manufacturer instructions (Wiener Lab, Argentina). This assay is based in Trinder reaction in which cholesterol in the sample is quantified by enzymatic hydrolysis of cholesterol esters (21).

### Lipid Analysis

Total lipids were extracted from the same number of macrophages with methanol/chloroform (2:1 v/v) as described by Bligh and Dyer (22). After extraction, lipids were dried and analyzed by thin layer chromatography on silica gel 60 F254 plates (Merck), using hexane/diethyl ether/acetic acid (75:25:1, v/v/v) as the developing solvent. Chemical staining with Cu-phosphoric was used for detection.

### Phenotypic Characterization by Flow Cytometry

Macrophages were centrifuged for 7 min at 1,200 rpm and then stained for 40 min at 4°C with fluorophore-conjugated antibodies FITC-anti-CD36 (clone 5-271), PerCP.Cy5.5-anti-CD14 (clone HCD14), PE-anti-CD163 (clone GHI/61), FITC-anti-CD206 (clone C068C2) (Biolegend), FITC-anti-HLA-DR (clone G46-6), or PE-anti-CD274 (clone MIH1) (BD Biosciences), and in parallel, with the corresponding isotype control antibody. After staining, the cells were washed with PBS 1×, centrifuged and analyzed by flow cytometry using FACSCalibur cytometer (BD Biosciences). The median fluorescence intensity were analyzed using FCS Express V3 software (*De Novo* Software, Los Angeles, CA, USA). The mean of the median fluorescence intensities (MFI) of the independent assays were used for comparisons between experimental conditions.

### Soluble Cytokine Determinations

The amounts of human TNF-α, IL-1β, IL-10, IL-6, IFN-γ, and IL-4 were measured by ELISA, according to manufacturers’ instructions kits (TNF-α, IL-4, and IFN-γ Ready-SET-Go!™ Kits from eBioscience; IL-10, IL-1β, and IL-6 ELISA MAX™ Deluxe Kits from Biolegend). The detection limit was 3 pg/ml for TNF-α; 8 pg/ml for IL-10 and IL-6; 6.25 pg/ml for IFN-γ and IL-4; and 15.6 pg/ml for IL-1β. Murine TNF-α and IL-10 were measured by ELISA, according to manufacturer’s instructions (OptEIA™ Set kits from BD Bioscience) with a detection limit at 30 pg/ml.

### Cytokine Depletions of PE

Tuberculosis-PE were incubated with 10 µg/ml of the following neutralizing antibodies for 1 h at 4°C for the specific depletion of IL-10 (αIL-10, clone 19F1; Biolegend), IL-6 (αIL-6, clone MQ2-13A5; BD Bioscience), IL-1β (αIL-1β, clone 8516.311; SIGMA), or TNF-α (αTNF-α, clone MAb1; Biolegend). Then, 100 µl/ml of Protein G Sepharose beads (Amersham) were added and incubated for 1 h at 4°C. Finally, TB-PE were centrifuged at 12,000 *g* to remove antibody-bead complexes and then filtered (0.22-µm pores) before use. IFN-γ depletion was performed by incubating TB-PE for 2 h in sterile 96-well plates that had been coated with the capture antibody provided by the Human IFN-γ ELISA Kit (BD Bioscience). In all cases, depletions were controlled by ELISA.

### Electron Microscopy

Macrophages exposed (or not) to TB-PE, and depleted (or not) for IL-10, were prepared for transmission electron microscopy study. For this purpose, cells were centrifuged at 6,000 rpm for 1 min, fixed in 1% glutaraldehyde dissolved in 0.1 M cacodylate buffer (pH 7); post-fixed in 2% osmium tetroxide; dehydrated with increasing concentrations of ethanol and gradually infiltrated with Epon resin (Pelco). Thin sections were contrasted with uranyl acetate and lead citrate (Electron Microscopy Sciences, Fort Washington, PA, USA) and examined with a FEI Tecnai transmission electron microscope (Hillsboro, OR, USA). For morphometry, 30 cells from each condition were randomly selected and digitalized at 40,000× magnification. Then the area of lipid electron dense vacuoles per cell were measured and compared in each experimental group by automated morphometry.

### Proliferation of Antimycobacterial CD4 T Cells Induced by Macrophages

We purified and maintained CD4 T cells from blood derived from healthy PPD^+^ donors as previously demonstrated (23). In parallel, we cultured autologous monocytes to generate macrophages and exposed them or not to TB-PE 20% v/v for 24 h. The next day, the medium was replaced, and macrophages were loaded with mycobacterial antigens by adding γ-irradiated Mtb at 2 bacteria to 1 macrophage ratio for further 24 h. Thereafter, the medium was replaced and autologous carboxyfluorescein succinimidyl ester (CFSE)-labeled CD4 T cells (Invitrogen) were added at a ratio of 10 lymphocytes to 1 macrophage for 5 days, as detailed previously (24). To determine IFN-γ production among proliferating CD4 T cells (CFSE^low^), brefeldin A (5 µg/ml; Sigma Chemical Co.) was added 4 h prior the end of the coculture to block cytokine secretion. Thereafter, CFSE-labeled lymphocytes were first stained with anti-CD4-PerCP-Cy5.5 mAbs (eBioscience), then fixed with 0.5% PFA for 15 min. Second, cells were permeabilized with 500 µl Perm2 (Becton Dickinson, Cockysville, MD, USA) for 10 min and incubated with anti-IFN-γ-PE mAb (Invitrogen, CA, USA). Cells were gated according to its FSC and side scatter (SSC) properties analyzed on FACScan (Becton Dickinson). In order to gate out dead lymphocytes, the gate of CD4 T cells with increased SSC and low FSC was excluded (25). Isotype matched controls were used to determine auto-fluorescence and non-specific staining. Analysis was performed using the FCS Express (*De Novo* Software) and results were expressed as percentages of IFN-γ^pos^/CFSE^low^/CD4^pos^ T cells induced by the different macrophage populations. To complement these results, the amounts of IFN-γ released in the supernatant throughout the coculture were determined by ELISA according to the manufacturer’s kit indications (BD Bioscience).

### Measurement of Bacterial Intracellular Growth in Macrophages by Colony Forming Unit (CFU) Assay

Macrophages exposed (or not) to TB-PE, were infected with H37Rv Mtb strain at a MOI of 1 bacteria/cell in triplicates. After 2 h, extracellular bacteria were removed by gently washing four times with pre-warmed PBS. At 4 h and days 3, 6, and 10, cells were lysed in 0.1% SDS and neutralized with 20% Bovine Serum Albumine in Middlebrook 7H9 broth. Serial dilutions of the lysates were plated in triplicate, onto 7H11-Oleic Albumin Dextrose Catalase (OADC, Difco) agar medium for CFU scoring at 21 days later.

### Western Blots

Macrophages were treated (or not) with TB-PE. Following the different experimental treatments, cells were lysed in ice-cold buffer consisting of 150 mM NaCl, 10 mM Tris, 5 mM EDTA, 1% SDS, 1% Triton X-100, 1% sodium deoxycholate, gentamicin/streptomycin, 0.2% azide plus a cocktail of protease inhibitors (Sigma-Aldrich). Lysates were incubated on ice for 3 h and cleared by centrifugation for 15 min at 14,000 rpm at 4°C. Protein concentrations were determined using the BCA protein assay (Pierce). Equal amounts of protein (40 µg) were then resolved on a 10% SDS-PAGE. Proteins were then transferred to Hybond-ECL nitrocellulose membranes (Amersham) for 2 h at 100 V and blocked with 1% BSA-0.05% Tween-20 for 1 h at room temperature. Membranes were then probed with primary anti-human ACAT (1:200 dilution, SOAT; Santa Cruz) or anti-human pY705-STAT3 (1:1,000 dilution, Cell Signaling Technology, clone D3A7) overnight at 4°C. After extensive washing, blots were incubated with a HRP-conjugated goat anti-rabbit IgG Ab (1:5,000 dilution; Santa Cruz Biotechnology) or HRP-conjugated goat anti-mouse IgG Ab (1:2,000 dilution; Santa Cruz Biotechnology) for 1 h at room temperature. Immunoreactivity was detected using ECL Western Blotting Substrate (Pierce). Protein bands were visualized using Kodak Medical X-Ray General Purpose Film. For internal loading controls, membranes were stripped by incubating in buffer consisting of 1.5% Glycine, 0.1% SDS, 1% Tween-20, pH 2.2 for 10 min twice, extensively washed and then reprobed with anti-β-actin (1:2,000 dilution; ThermoFisher, clone AC-15) or anti-STAT3 Ab (1:1,000 dilution; Cell Signaling Technology, clone D1A5). Results from Western blot were analyzed by densitometric analysis (Image J software).

### Immunostaining

Macrophages were plated on glass coverslips and were treated with or without 20% v/v of TB-PE for 24 h. Cells were fixed with PFA 4% for 20 min at room temperature and then PFA was quenched with 50 mM NH_4_Cl for 2 min. Cells were rinsed in PBS once and then were labeled with 1 µg/ml of BODIPY 493/503 (Life technologies) for 15 min before permeabilization with PBS-Triton X-100 0.1% for 10 min. Cells were then incubated with PBS-BSA 3% w/v for 30 min prior to overnight incubation at 4°C with primary anti-human pY705-STAT3 (dilution 1/100, Cell Signaling Technology, Clone D3A7). Cells were then washed and incubated with Goat anti-Mouse IgG, AlexaFluor 555 (dilution 1/1,000, Cell Signaling Technology) for 1 h at room temperature. Cells were extensively washed and then incubated for 10 min with DAPI in PBS-BSA 1% (500 ng/ml, Sigma-Aldrich). Finally, slides were mounted and visualized with a Leica DM-RB fluorescence microscope.

### Mice

Wild-type (WT) BALB/c and IL-10 knockout (IL-10 KO) (C.129P2(B6)-IL-10 tm1Cgn/J) BALB/c male mice (8–12 weeks old), were obtained from the Leloir Institute Foundation. Animals were bred and housed in accordance with the guidelines established by the Institutional Animal Care and Use Committee of Institute of Experimental Medicine (IMEX)-CONICET-ANM. All animal procedures were shaped to the principles set forth in the Guide for the Care and Use of Laboratory Animals (26).

### Generation of BMDMs

Femurs and tibia from WT and IL-10 KO mice were removed after euthanasia and the bones were flushed with RPMI-1640 medium by using syringes and 25-Gauge needles. The cellular suspension was centrifuged and the red blood cells were removed. The BMDMs were obtained by culturing the cells with RPMI-1640 medium containing l-glutamine, pyruvate, β-mercaptoethanol (all from Sigma-Aldrich), 10% FCS and 20 ng/ml of murine recombinant M-CSF (Biolegend) at 37°C in a humidified incubator for 7–8 days. Differentiated BMDMs were re-plated into 24-well tissue culture plates in complete medium prior to cell stimulation with Mtb lipids for 24 h. Alternatively, recombinant murine IL-10 (Peprotech) at 10 ng/ml was added to the cultures for 24 h.

### Statistical Analysis

All values are presented as mean and SEM of a number of independent experiments. Independent experiments are defined as those performed with macrophages derived from monocytes isolated independently from different donors. As most of our datasets did not pass the normality tests, non-parametric tests were applied. Comparisons between more than two paired data sets were made using the Friedman test followed by Dunn’s Multiple Comparison Test. Comparisons between two paired experimental conditions were made using the two-tailed Wilcoxon Signed Rank. Correlation analyses were determined using the Spearman’s rank test. For all statistical comparisons, a *p* value < 0.05 was considered significant.

## Results

### Tuberculous PE Fluids Induce Lipid Bodies Accumulation in Macrophages

**Figure 1:**
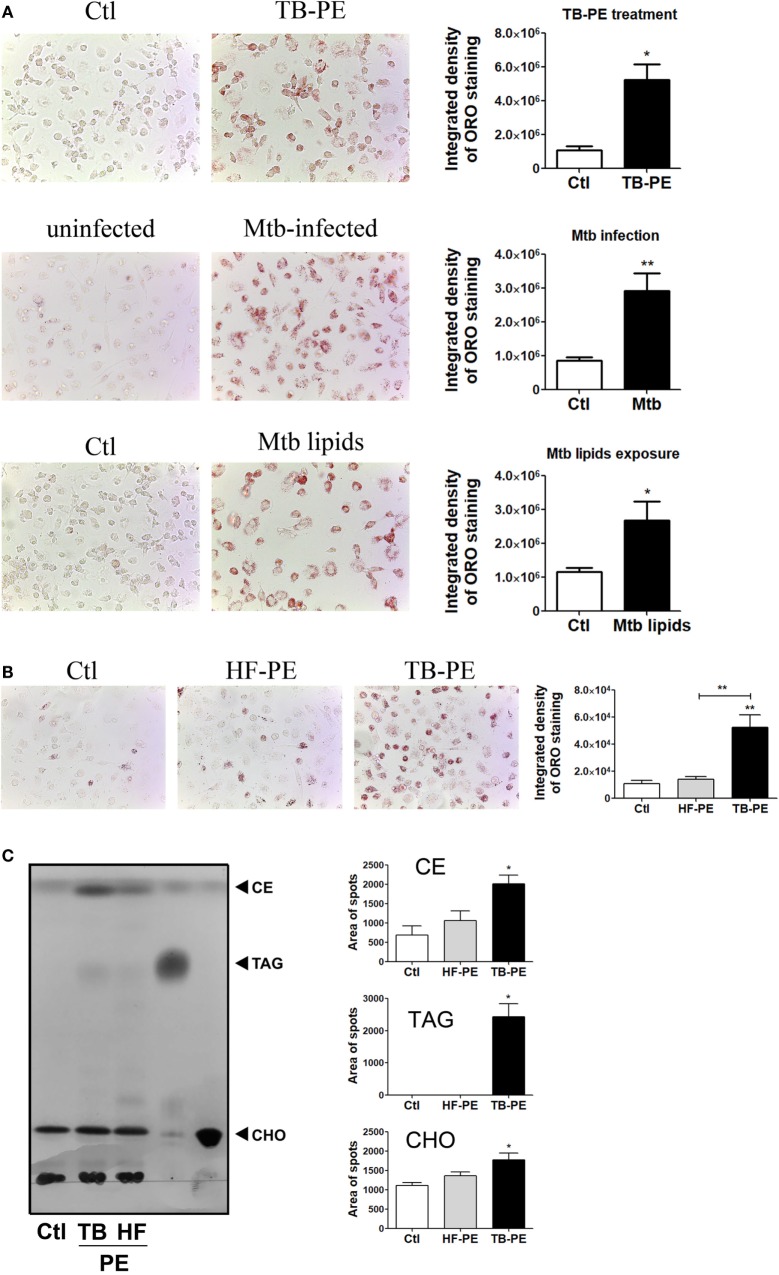

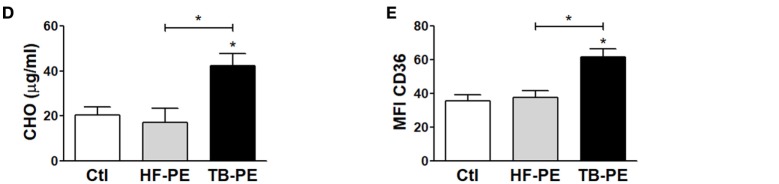
Tuberculous pleural effusions (TB-PE) induce lipid bodies accumulation in macrophages. **(A)** Human monocyte-derived macrophages (MDM) were treated either with TB-PE or with *Mycobacterium tuberculosis* (Mtb) lipids, or infected with Mtb H37Rv for 24 h and then stained with Oil Red O (ORO). Representative images are shown in left panels (40× magnification) and the integrated density of ORO staining is shown in right panels. Values are expressed as means ± SEM of six independent experiments, considering five microphotographs per experiment. Wilcoxon signed rank test: *p < 0.05. **(B)** MDM were treated with TB-PE or PE from heart failure patients (HF-PE) and stained with ORO (*n* = 5). **(C)** Left panel shows a representative thin layer chromatographic analysis of lipids from MDM treated or not with either TB-PE or HF-PE. Total lipids were extracted from untreated MDM (lane 1), TB-PE-treated MDM (lane 2), HF-PE-treated MDM (lane 3), and the standard lipids triacylglycerol (TAG, lane 4) and free cholesterol (CHO, lane 5). Cholesterol esters (CE) are also indicated. Right panels depict the area of spots for CE, TAG, and CHO in control and TB-PE or HF-PE treated MDM (*n* = 4). **(D)** Intracellular total cholesterol (CHO) content in MDM exposed or not to TB-PE or HF-PE measured by an enzymatic method (*n* = 5). **(E)** Mean fluorescence intensity (MFI) of CD36 cell-surface expression in MDM exposed or not to TB-PE or HF-PE measured by flow cytometry (*n* = 5). **(B–E)** Friedman test followed by Dunn’s Multiple Comparison Test: *p < 0.05; **p < 0.01 for TB-PE vs Ctl or as depicted by lines.

It has been demonstrated that Mtb induces a lipid-rich foam cell phenotype in host macrophages (6–8, 27), possibly *via* TLR2 and TLR6 activation (28). Moreover, merely isolated lipids from Mtb were able to induce the foamy phenotype (7, 27). Based on this knowledge, we aimed to determine whether soluble factors found in physiological samples derived from active TB patients could promote the generation of FM. To this end, we treated human M-CSF-driven macrophages with cell-free preparations of PE derived from patients with tuberculosis (TB-PE) in order to mimic a genuine microenvironment derived during Mtb infection. According to the pattern of staining of neutral lipids with ORO, we observed that TB-PE treatment induced lipid bodies accumulation in macrophages to the same extent as Mtb infection or exposure to Mtb-derived lipids (Figure 1A). We also demonstrated that the formation of lipid bodies was specific for TB-PE treatment in comparison to PE from patients with heart failure (HF-PE) (Figure 1B). Moreover, biochemical analysis of the lipids recovered from macrophages identified higher abundance of cholesteryl esters and triacylglycerols in TB-PE-treated macrophages in comparison to control or HF-PE-treated macrophages (Figure 1C). Additionally, unlike HF-PE, the treatment with TB-PE resulted in an increase of the total intracellular cholesterol content (Figure 1D) and a twofold increase of CD36 expression (Figure 1E). Therefore, soluble factors released during pleural Mtb infection induced lipid body accumulation in human macrophages that typically confirm the FM phenotype.

### FM Induced by Tuberculous PE Display Immunosuppressive Properties

**Figure 2:**
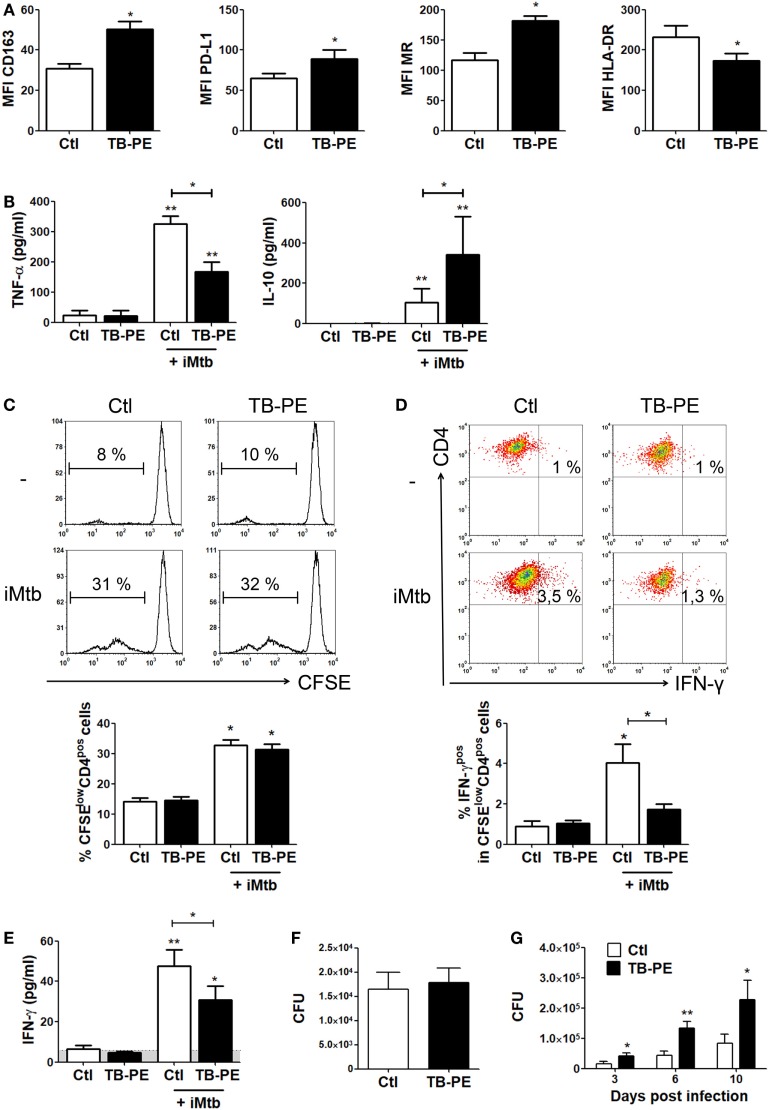
Macrophages treated with tuberculous pleural effusion (TB-PE) display immunosuppressive properties. **(A)** Mean fluorescence intensity (MFI) of CD163, PDL-1, MR, and HLA-DR measured by flow cytometry in human monocyte-derived macrophages (MDM) exposed or not to TB-PE (*n* = 5). Wilcoxon signed rank test: *p < 0.05. **(B)** Levels of secreted IL-10 and TNF-α by MDM exposed or not to TB-PE in response to irradiated *Mycobacterium tuberculosis* (iMtb) measured by ELISA (*n* = 5). Friedman test followed by Dunn’s Multiple Comparison Test: *p < 0.05; **p < 0.01, for iMtb-stimulated vs unstimulated, or as indicated in the graph. **(C–E)** MDM from healthy PPD^+^ donors exposed or not to TB-PE for 24 h, were stimulated or not with iMtb for 24 h and then used as antigen presenting cells (APC) in autologous proliferation assays. Cocultures were performed with autologous carboxyfluorescein succinimidyl ester (CFSE)-labeled CD4 T cells at a ratio of 10 T cells: 1MDM. **(C)** Representative histograms showing CFSE labeling in CD4^pos^ T cells activated by macrophages exposed (or not) to TB-PE, loaded (or not) with iMtb. Right panel shows the quantification of the percentages of proliferating CD4^pos^ T cells (CFSE^low^/CD4^pos^ cells) in each condition. Loaded vs unloaded with iMtb: *p ≤ 0.05 (*n* = 4). **(D)** Representative dot plots showing CD4 and IFN-γ expression among CFSE^low^/CD4^pos^ T cells activated by MDM exposed (or not) to TB-PE, loaded (or not) with iMtb. Right panel shows the quantification of the percentages of proliferating IFN-γ-producing CD4^pos^ T cells (IFN-γ^pos^/CFSE^low^/CD4^pos^ T cells) in each condition (*n* = 4). Friedman test followed by Dunn’s Multiple Comparison Test: *p < 0.05, for iMtb-stimulated vs unstimulated, or as indicated in the graph. **(E)** The amounts of IFN-γ released throughout the coculture were determined by ELISA. Friedman test followed by Dunn’s Multiple Comparison Test: *p < 0.05; **p < 0.01, for iMtb-stimulated vs unstimulated, or as indicated in the graph. **(F)** Bacillary loads in MDM treated (or not) with TB-PE, washed, and infected with *Mycobacterium tuberculosis* (Mtb) strain H37Rv for 4 h (*n* = 6). Wilcoxon signed rank test. **(G)** Intracellular colony forming units were determined at different time points in MDM treated with TB-PE for 24 h, washed and infected with Mtb (*n* = 10). Wilcoxon signed rank test: *p < 0.05; **p < 0.01; ***p < 0.001 for TB-PE treated vs Ctl.

In order to assess whether the differentiation into FM induced by TB-PE may have a negative impact on the development of the antimycobacterial response, we determined phenotype and functions of TB-PE-induced FM. As observed in Figure 2A, macrophages treated with TB-PE displayed a higher expression of anti-inflammatory markers such as CD163, mannose receptor or CD206 (MRC1), and PD-L1, and a lower expression of the MHC class II cell-surface receptor, HLA-DR. In accordance with the acquisition of this anti-inflammatory profile, TB-PE-induced FM secreted higher levels of IL-10 and lower levels of TNF-α upon stimulation with irradiated *Mycobacterium tuberculosis* (iMtb) than untreated cells (Figure 2B). In order to assess the effect of TB-PE treatment on the presentation of mycobacterial antigens, macrophages were treated with or without TB-PE for 24 h, washed and stimulated with iMtb for further 24 h. Thereafter, cells were washed and cocultured with autologous CFSE-labeled CD4 T cells derived from healthy PPD positive subjects for 5 days. Based on the CFSE labeling of CD4 T cells, while TB-PE-treated macrophages did not induce differential levels of proliferation of antimycobacterial CD4 T cells (Figure 2C), they promoted differential distribution of the IFN-γ producing clones (Figure 2D). In particular, those macrophages treated with TB-PE induced lower percentages of IFN-γ producing clones (Figure 2D) and a lower release of IFN-γ (Figure 2E), as compared to untreated macrophages in response to iMtb stimulation. Of note, IL-4 and IL-17 contents were undetectable in these assays (data not shown). Therefore, the activation of Ag-specific IFN-γ-producing CD4 T cells was impaired when macrophages were exposed to TB-PE, suggesting that the accumulation of lipid bodies is accompanied by a reduced capacity to activate antimycobacterial Th1 cells.

Next, we assessed whether the treatment with TB-PE had an impact on the control of the bacillary load. To accomplish this, macrophages were treated with TB-PE for 24 h, washed and infected with Mtb. Although no differences were observed in the uptake of the mycobacteria as judged by the scoring of the CFU at 4 h post-infection (Figure 2F), a significant increase in the bacillary load was observed in TB-PE-treated macrophages at later time points (Figure 2G). Therefore, FM induced by TB-PE are more susceptible to Mtb intracellular growth.

### IL-10 Promotes Accumulation of Lipid Bodies in Macrophages under TB-PE Treatment

**Figure 3:**
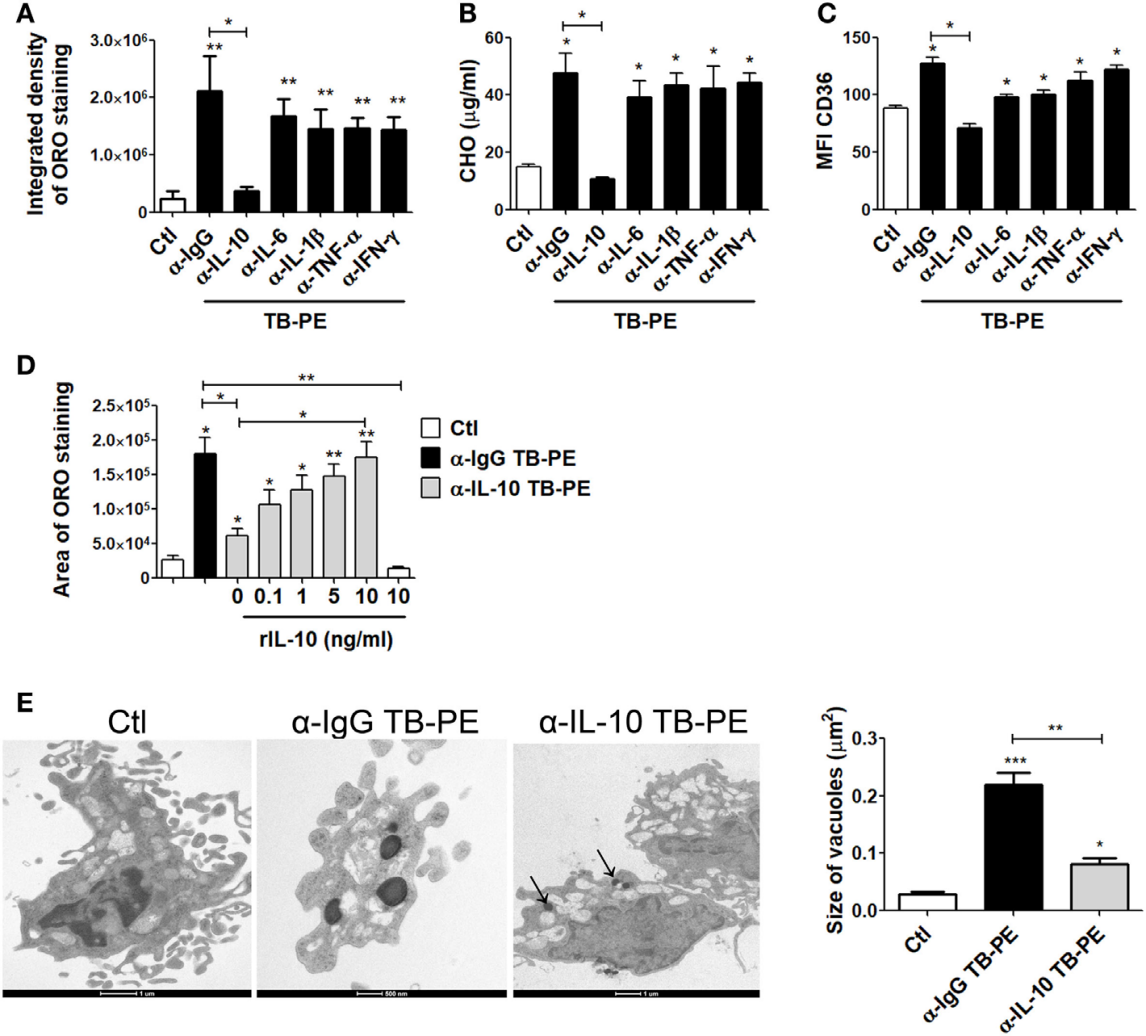
Interleukin-10 (IL-10) promotes lipid bodies accumulation in macrophages treated with tuberculous pleural effusion (TB-PE). **(A–C)** Human monocyte-derived macrophages (MDM) were treated with TB-PE depleted or not for different cytokines for 24 h and **(A)** lipid bodies’ content was measured by Oil Red O (ORO) staining (*n* = 4). **(B)** Intracellular cholesterol (CHO) content measured by an enzymatic method (*n* = 4) was determined. **(C)** Mean fluorescence intensity (MFI) of CD36 cell-surface expression was measured by flow cytometry (*n* = 4). **(D)** MDM were treated (or not) with TB-PE depleted or not of IL-10 in the presence of different amounts of recombinant human IL-10 (rIL-10) for 24 h and lipid bodies content was measured by ORO staining (*n* = 6). **(E)** Representative electron microscopy micrographs of MDM treated with TB-PE depleted or not of IL-10. White asterisks indicate large electron dense osmiophilic vacuoles and arrows point small sized ones. Morphometric study of the area per vacuole in each condition is shown (*n* = 14). **(A–E)** Friedman test followed by Dunn’s Multiple Comparison Test: *p < 0.05; **p < 0.01; ***p < 0.001 for experimental condition vs Ctl or as depicted by lines.

In order to evaluate the host factors involved in promoting the accumulation of lipid bodies by TB-PE, we depleted different cytokines known to be highly present in this fluids (29, 30), including IL-10, TNF-α, IL-1β, IL-6, and IFN-γ. We then evaluated the ability of these depleted-PE to induce the foamy phenotype in macrophages. The depletion of each individual cytokine was confirmed by ELISA (Figure S1A in Supplementary Material). Interestingly, only the depletion of IL-10 from TB-PE was capable of preventing lipid bodies accumulation (Figure 3A). This result was also confirmed by labeling the cells with Bodipy staining (Figure S1B in Supplementary Material). In addition, macrophages exposed to IL-10-depleted TB-PE showed a reduction of intracellular cholesterol content and CD36 cell-surface expression in comparison to those cells treated with non-depleted or depleted of any other cytokines (Figures 3B,C). In line with these results, the addition of recombinant IL-10 to the IL-10-depleted TB-PE restored the acquisition of the foamy phenotype in a dose-dependent manner (Figure 3D). Of note, the addition of IL-10 in the absence of TB-PE did not induce the foamy phenotype (Figure 3D). Thereafter, we determined the presence of the lipidic vacuoles by electron microscopy in macrophages treated with TB-PE depleted (or not) of IL-10. As it is shown in Figure 3E, while untreated macrophages showed numerous pseudopodia and empty cytoplasmic vacuoles, TB-PE-treated macrophages displayed electron dense lipid osmiophilic vacuoles, which correspond to lipid bodies. Interestingly, macrophages treated with IL-10-depleted TB-PE showed smaller-sized lipidic vacuoles than those exposed to non-depleted TB-PE. These results indicate that the IL-10 present in TB-PE is a key host factor promoting the lipid-laden phenotype in the presence of exogenous lipids.

### IL-10 Levels Correlate with FM Abundance in PE

**Figure 4:**
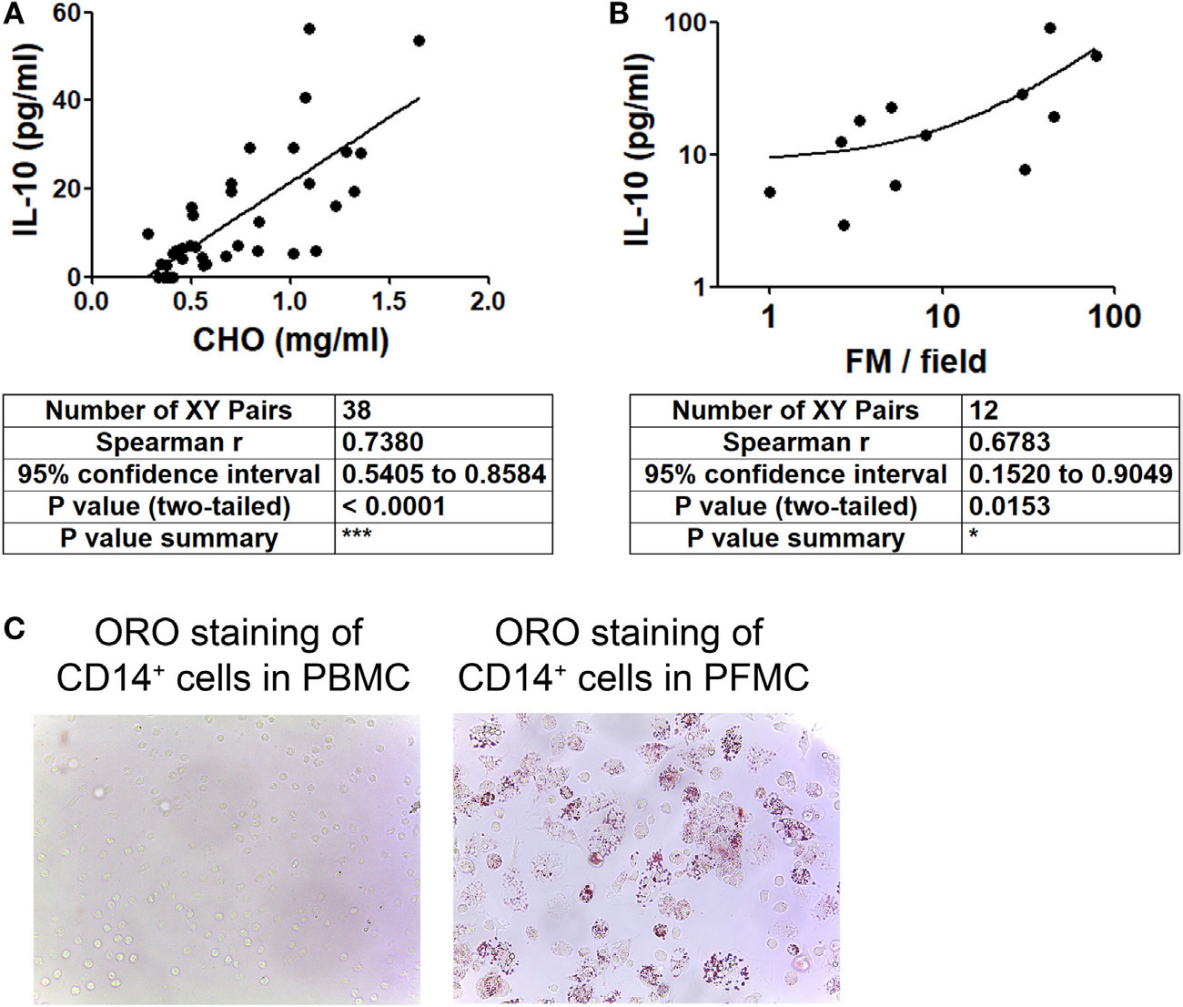
Cholesterol levels and foamy macrophages (FM) are associated with interleukin-10 (IL-10) amounts present in tuberculous pleural effusion (TB-PE). **(A,B)** Correlation analysis between the levels of IL-10 and **(A)** the cholesterol content (*n* = 38) or **(B)** the number of lipid-laden CD14^+^ cells within the pleural fluids mononuclear cells (PFMC) (*n* = 12) found in individual preparations of TB-PE. In B log-10 scale for both axes is used. Linear regression lines are shown. Spearman’s rank test. **(C)** Representative image of ORO staining of CD14^+^ cells isolated from paired peripheral blood mononuclear cells (PBMC) and PFMC is shown (40× magnification).

In order to evaluate whether IL-10 level is associated to the acquisition of the foamy phenotype in the course of a Mtb natural infection, we assessed the levels of IL-10 and total cholesterol in individual preparations of TB-PE, and the numbers of FM found within the pleural fluids mononuclear cells (PFMC). We found that levels of IL-10 and total cholesterol were positively correlated (Figure 4A), unlike other cytokines such as IFN-γ, IL-6, TNF-α, and IL-1β (Figure S2 in Supplementary Material). Noticeably, there was also a positive correlation between the level of IL-10 and the number of FM among the PFMC (Figure 4B). Moreover, lipid-laden CD14^+^ cells were found in the pleural compartment but not in paired peripheral blood (Figure 4C). These results support the idea that IL-10 potentiates the acquisition of the foamy program in human macrophages in the context of a natural infection.

### IL-10 Deficiency Prevents the Foamy Phenotype in BMDM

**Figure 5:**
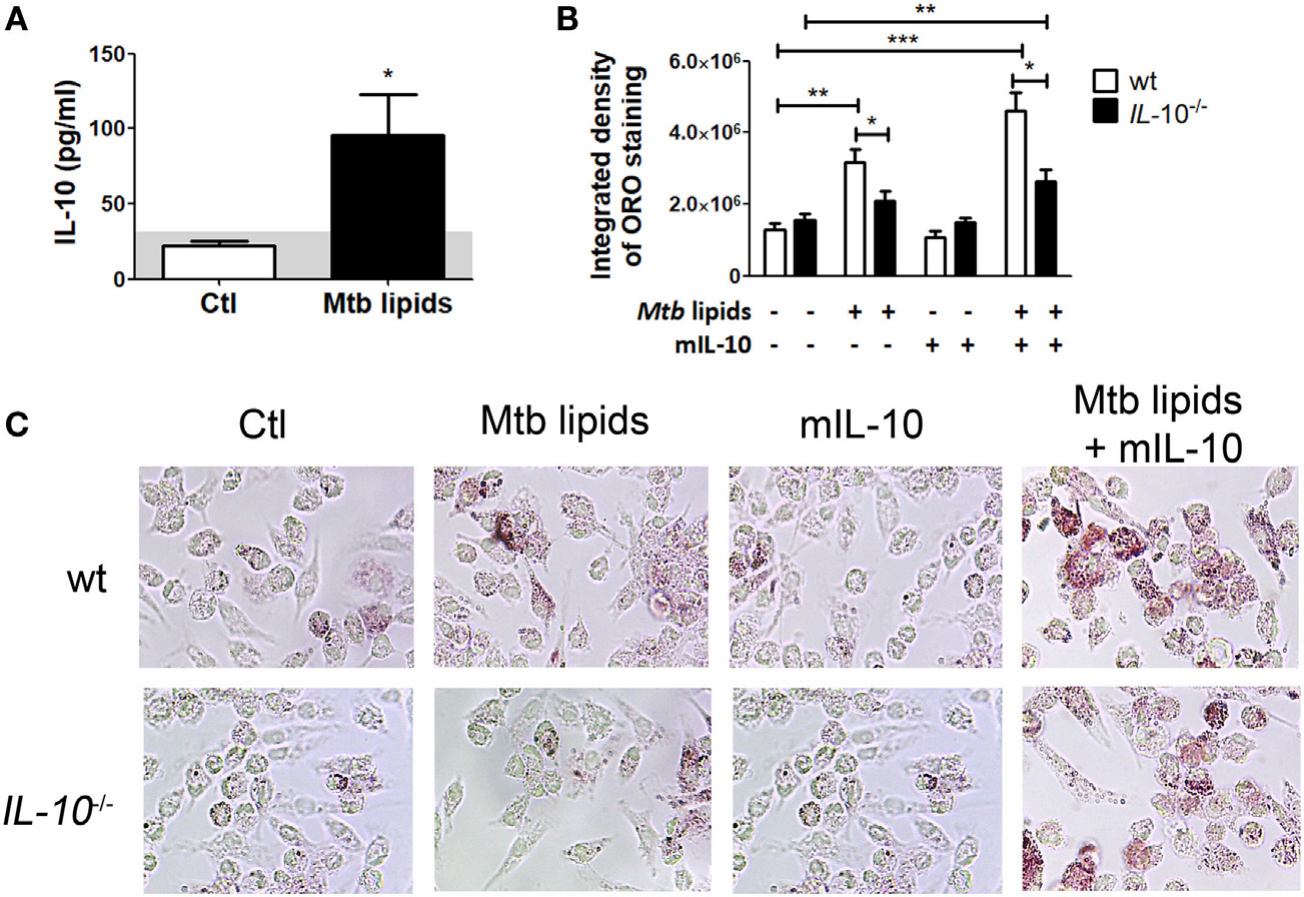
Interleukin-10 (IL-10) deficiency reduces lipid bodies accumulation in bone marrow-derived mouse macrophages (BMDM) in response to *Mycobacterium tuberculosis* (Mtb) lipids. **(A)** BMDM from wt mice were treated or not with Mtb lipids for 24 h and the levels of secreted IL-10 were measured by ELISA (*n* = 5) Wilcoxon signed rank test: *p < 0.05. **(B,C)** BMDM from wt or *IL-10*^−/−^ mice were treated or not with Mtb lipids in the presence or not of recombinant murine IL-10 for 24 h and then stained with Oil Red O (ORO). The integrated density of ORO staining **(B)** and representative images **(C)** are shown (*n* = 6). Friedman test followed by Dunn’s Multiple Comparison Test: *p < 0.05; **p < 0.01; ***p < 0.001 as depicted by lines.

To confirm the role of IL-10 in the differentiation of FM, we evaluated whether BMDM derived from IL-10-knock out (KO) mice could indeed become FM after the treatment with lipids from Mtb. First, we determined IL-10 production in M-CSF-derived BMDM from WT mice stimulated with mycobacterial lipids for 24 h. As shown in Figure 5A, unlike the undetectable level of TNF-α (data not shown), BMDM secreted IL-10 in response to mycobacterial lipid-stimulation. Second, we compared whether BMDM derived from WT or IL-10-KO mice differed in their propensity to accumulate lipid bodies in response to Mtb-derived lipids. As illustrated in Figures 5B,C, IL-10-deficiency prevented the foamy phenotype induced by Mtb lipids, which in turn could be partially reverted by the addition of exogenous IL-10. In agreement with our previous results, exogenous IL-10 did not induce the foamy phenotype in the absence of the source of lipids in BMDM. These results obtained in murine macrophages confirm those obtained in human TB-PE-treated macrophages, demonstrating the key role of IL-10 in favor of the differentiation program toward foamy cells.

### The IL-10/STAT3 Axis Is Involved in the FM Differentiation Induced by TB-PE

**Figure 6:**
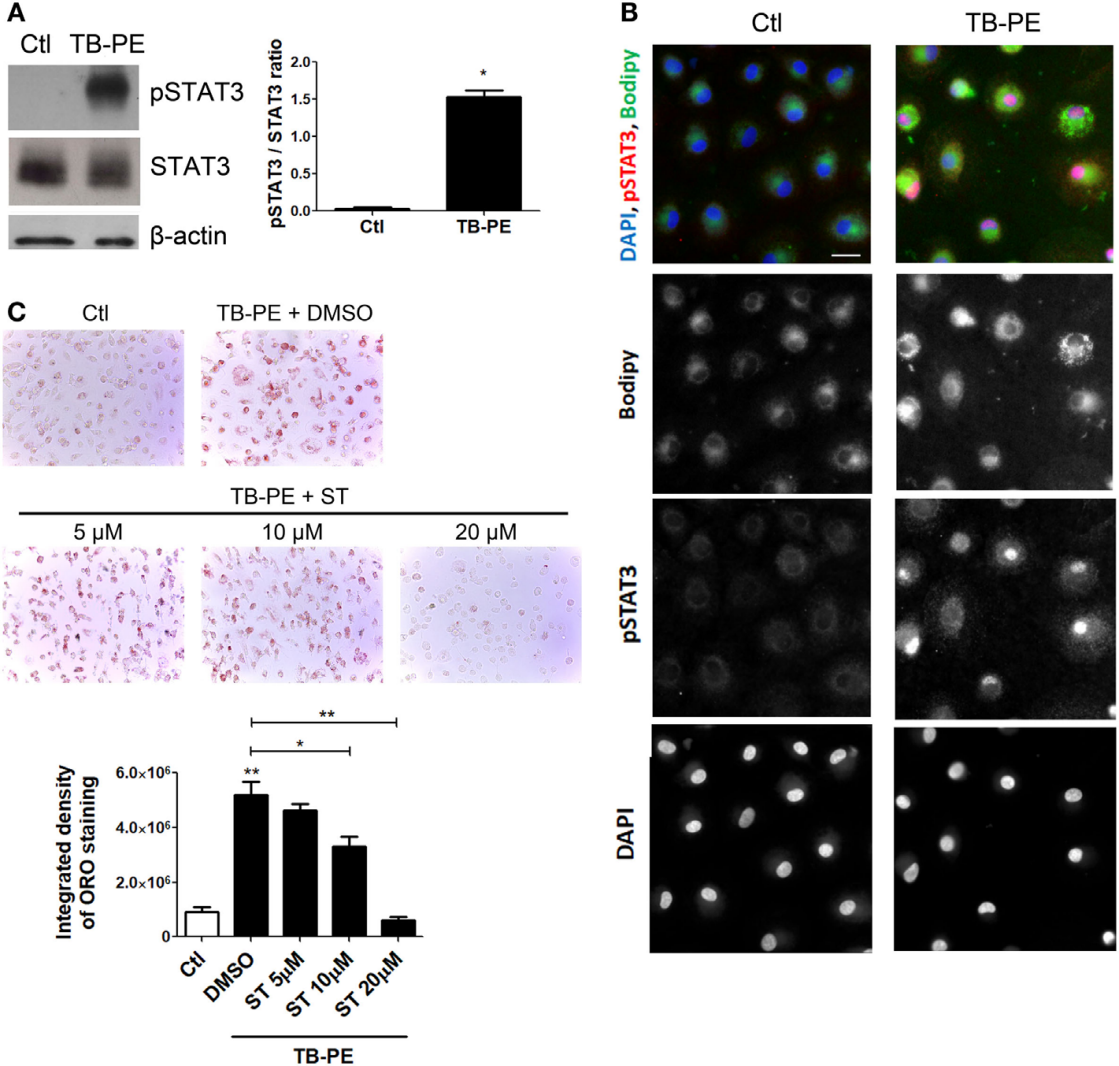
Signal transducer and activator of transcription 3 (STAT3) activation enhances lipid bodies accumulation induced by treatment of macrophages with tuberculous pleural effusion (TB-PE). **(A)** Analysis of p705-STAT3, STAT3, and β-actin protein expression level by western Blot (left panel) and quantification (right panel; *n* = 4) in human monocyte-derived macrophages (MDM) treated with TB-PE for 24 h. Wilcoxon signed rank test: *p < 0.05 **(B)** MDM were treated or not with TB-PE for 24 h and then were fixed, labeled with Bodipy 493/503 (green), permeabilized, and stained for p705-STAT3 (Red) and DAPI (blue). Representative images are shown. Scale bar, 10 µm. **(C)** MDM were treated or not with different concentrations of Stattic (STAT3 inhibitor, ST) for 2 h and then exposed or not to TB-PE for 24 h. Lipid bodies’ content was determined by Oil red O (ORO) staining (*n* = 6). Representative images are shown and the integrated density of ORO staining is shown. Friedman test followed by Dunn’s Multiple Comparison Test: *p < 0.05; **p < 0.01 for TB-PE treated vs Ctl, or as depicted by lines.

Considering the role of IL-10 in promoting the differentiation of macrophages into FM, and that STAT3 is a pivotal transcriptional factor induced by IL-10 (31, 32), we studied the contribution of STAT3 activation in the induction of the foamy phenotype by measuring its phosphorylated form (pSTAT3). First, we determined that STAT3 was activated in TB-PE-treated macrophages detecting its phosphorylated form by western blot and immunofluorescence microscopy (Figures 6A,B). As depicted in Figure 6B FM induced by TB-PE showed nuclear localization of STAT3 phosphorylated on tyrosine 705, reflecting STAT3 activation. Importantly, pharmacological inhibition of STAT3 with Stattic (or with cucurbitacin I) prevented the accumulation of lipid bodies in macrophages in a dose-dependent manner (Figure 6C; Figures S3A,B in Supplementary Material). Therefore, the enhancement of FM differentiation driven by IL-10 is mediated by STAT3.

### IL-10 Enhances ACAT Expression in TB-PE-Treated Macrophages Leading to FM Differentiation

**Figure 7:**
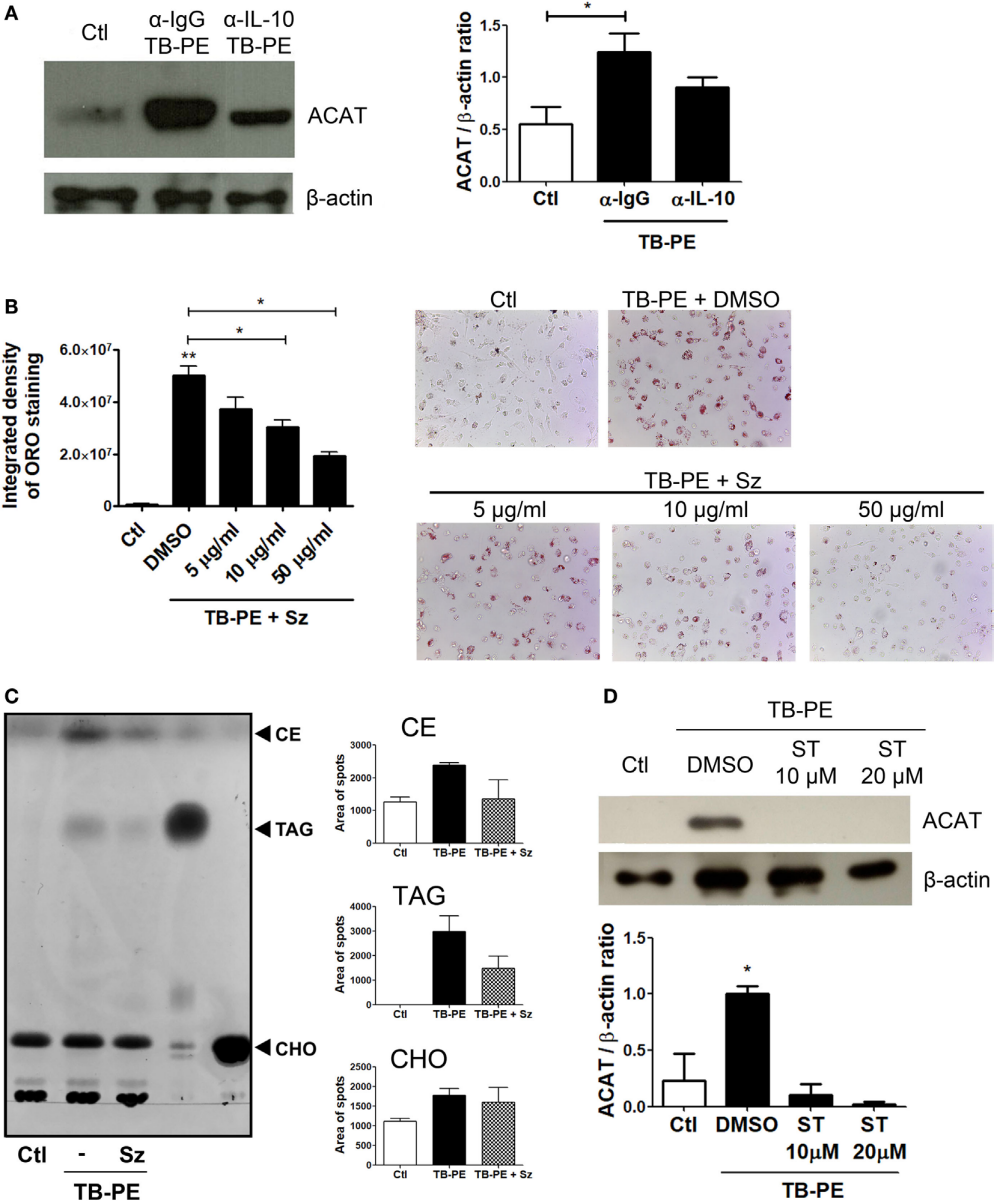
The interleukin-10 (IL-10)/signal transducer and activator of transcription 3 (STAT3) axis promotes foamy macrophage formation through ACAT upregulation. **(A)** Analysis of ACAT and β-actin protein expression level by western Blot (left panel) and quantification (right panel; *n* = 5) in human monocyte-derived macrophages (MDM) treated with tuberculous pleural effusion (TB-PE) for 24 h depleted or not for IL-10. **(B)** MDM were treated or not with different concentrations of Sandoz (Sz, ACAT inhibitor) for 1 h, exposed or not to TB-PE for 24 h, and then stained with Oil Red O (ORO). The Integrated density and representative images are shown (*n* = 6). **(A,B)** Friedman test followed by Dunn’s Multiple Comparison Test: *p < 0.05; **p < 0.01 for TB-PE treated vs Ctl, or as depicted by lines. **(C)** Thin layer chromatographic analysis of lipids from MDM treated or not with TB-PE in the presence or not of Sz. Total lipids were extracted from untreated MDM (lane 1), TB-PE-treated MDM (lane 2), TB-PE-treated MDM in the presence of Sz (lane 3), and the standard lipids triacylglycerol (TAG, lane 4) and cholesterol (CHO, lane 5). Cholesterol esters (CE) are also indicated. Right panels depict the area of spots for CE, TAG, and CHO in control and TB-PE in the presence or not of Sz treated MDM (*n* = 2). **(D)** Analysis of ACAT and β-actin protein expression level by Western Blot (left panel) and quantification (right panel; *n* = 3) in MDM treated or not with different concentrations of Stattic (ST) for 2 h and then exposed or not to TB-PE for 24 h. Friedman test followed by Dunn’s Multiple Comparison Test: *p < 0.05 for TB-PE treated vs Ctl.

In order to elucidate the mechanism by which IL-10 promotes FM differentiation, we assessed the expression of ACAT, which is central for the biogenesis of lipid bodies by converting free- into esterified-cholesterol that are eventually packed inside the lipid droplets (33). We first showed that the foamy phenotype induced by TB-PE was dependent on ACAT activity, as judged both by the increase of ACAT expression after TB-PE treatment (Figure 7A), and by the blocking of the accumulation of lipid bodies in the presence of Sandoz, a specific inhibitor of ACAT (Figure 7B). Noticeably, the inhibition of ACAT lead to reduced amounts of both cholesteryl esters and triacylglycerols in TB-PE-treated macrophages (Figure 7C), confirming the involvement of ACAT in FM formation upon TB-PE treatment. We then assessed ACAT expression in IL-10-depleted TB-PE and we found that its expression was reduced in the absence of IL-10 (Figure 7A). In agreement with this result, ACAT induction by TB-PE was abolished when STAT3 activity was inhibited (Figure 7D). Therefore, the IL-10/STAT3 axis activated by TB-PE enhances FM differentiation through the upregulation of ACAT expression, leading to an increased biogenesis and accumulation of lipid bodies.

**Figure 8:**
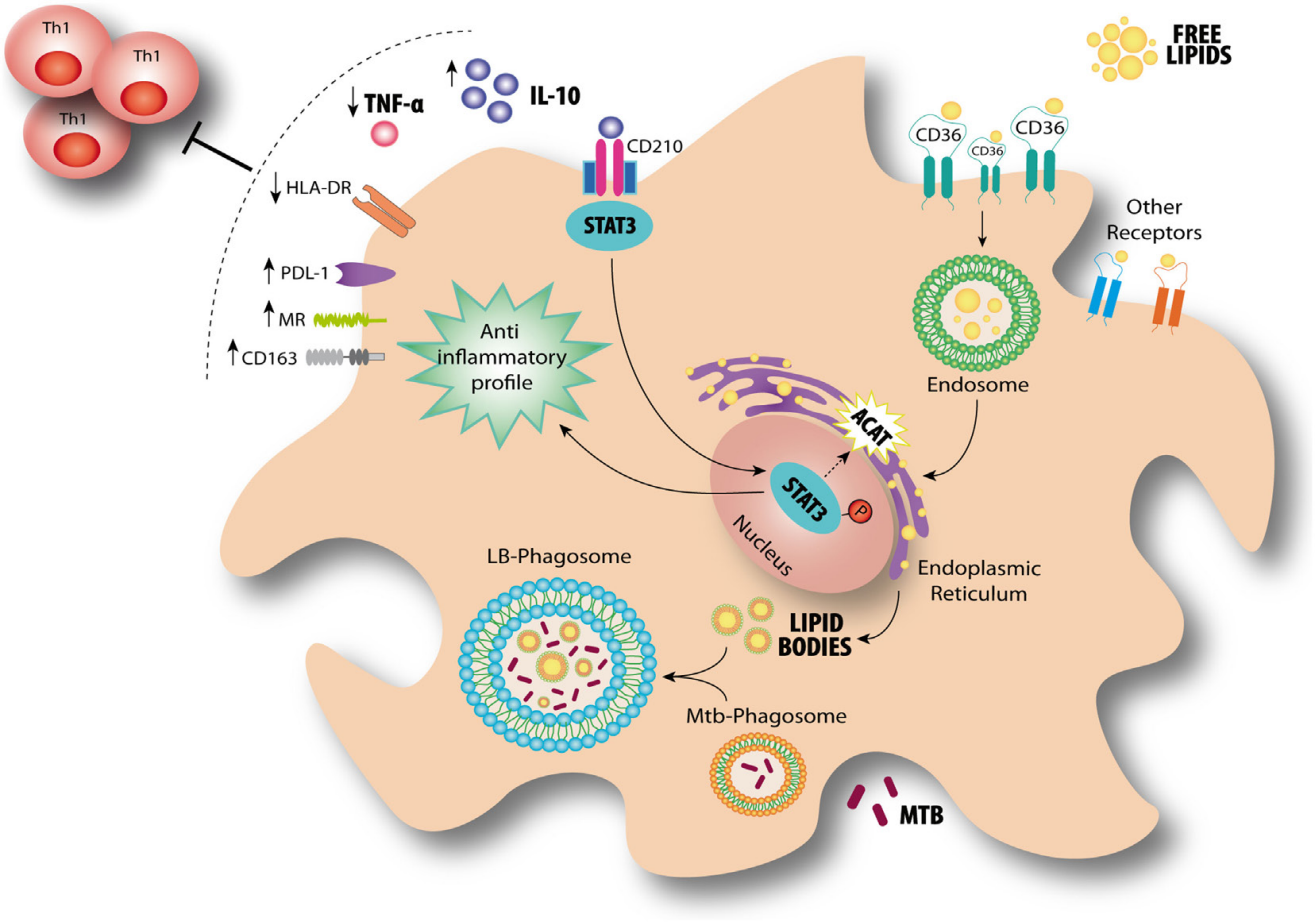
Interleukin-10 (IL-10) promotes foamy macrophage (FM) formation in an IL-10/signal transducer and activator of transcription 3 (STAT3)-dependent manner in tuberculosis (TB). According to our results, FM induced by pleural effusion from TB patients display immunosuppressive properties such as high IL-10 release, low TNF-α production, impaired Th1 activation, and high bacillary loads. At the same time, lipid bodies’ accumulation is enhanced by IL-10/STAT3 axis through an increase of CD36 cell-surface expression levels and ACAT expression. It has been demonstrated that *Mycobacterium tuberculosis* (Mtb)-containing phagosomes migrate toward these lipid bodies and engulf them (7). This mechanism ensures protection from microbicidal pathways and allows persistence of the bacilli inside host. Our results provide additional mechanisms by which the environment created by the infection context drives the FM differentiation even in the absence of the pathogen.

Based on our findings, we propose a model for the modulation of FM in the context of a physiologically relevant microenvironment promoted by Mtb infection for which the axis IL-10/STAT3 induces the accumulation of lipid bodies *via* ACAT upregulation. This in turn is accompanied by an increase of CD36 and the acquisition of immunosuppressive properties, such as a reduced induction of antimycobacterial Th1 clones, an enhanced production of IL-10 and a more permissive phenotype for bacillary growth (Figure 8).

## Discussion

Tuberculosis, as a chronic condition, entails the establishment of extensive metabolic remodeling in both host and pathogen. One of the consequences of this adaptation is the formation of FM. Since FM have been associated with the bacilli persistence and tissue pathology (6, 8, 12, 27, 34), we aimed to determine which host factors may contribute to enlarge the pool of FM in TB. In this sense, we used the acellular fraction of TB-PE to mimic those soluble factors released locally during Mtb infection. Although pleural disease due to *Mtb* is generally categorized as extra-pulmonary, there is an intimate anatomic relationship between the pleura and the pulmonary parenchyma (35, 36). Current literature supports the notion that TB-PE is the consequence of a direct local infection with a cascade of events, including an immunological response, instead of the result of a pure delayed hypersensitivity reaction, as previously thought (37). Even though we cannot state that macrophages infiltrating the pleural cavity will reproduce with fidelity those macrophages in the infected alveolar space or lung interstitial tissue, we can affirm that TB-PE represents microenvironment from a human respiratory cavity that is impacted by the infection. To our knowledge, this is the first time that such a complex but physiologically relevant human sample has been used in order to study the biology of FM. Using this *in vitro* model, we demonstrated that the acellular fraction of TB-PE induces the accumulation of lipid bodies in human macrophages. Moreover, our finding was validated by the detection of lipid-laden CD14^+^ cells isolated directly from the mononuclear cells of the PE, providing physiological relevance to our *in vitro* model. Taking into account that tuberculous PE has, if any, very few bacilli content (38), and that it displays high cholesterol content, we infer that in our model the source of lipids feeding the lipid bodies in the macrophages are likely host-derived components instead of mycobacterial ones.

Although lipid bodies were qualified as passive organelles involved in lipid storage, it became clear that these organelles play a central role in several inflammatory (e.g., atherosclerosis) or chronic infectious diseases (e.g., TB) (8, 13). In this work, we provide evidence that FM differentiation induced by TB-PE is potentiated by IL-10 in association with the acquisition immunosuppressive properties, impairing the activation of antimycobacterial Th1 clones, producing the anti-inflammatory cytokine IL-10 and bearing higher bacillary loads. This is in line with previous reports characterizing the immunosuppressive profile in macrophages activated by the IL-10/STAT3 signaling pathway (39, 40). In this study, we show that FM formation depends (albeit partially) on the IL-10/STAT3 axis, and thus establishing an association between both processes (accumulation of lipid bodies and immunosuppression). Within this context, we observed that both processes are enhanced by the IL-10/STAT3 signaling pathway, arguing for likelihood of two independent outcomes emanating from the same signaling axis (Figure 8). Of note, it has been reported that the synthetic glucocorticoid dexamethasone can upregulate ACAT expression promoting formation of FM (41). Although there are extensive reports demonstrating transcriptional interactions between STAT3 and glucocorticoids leading to repression or synergism of target genes (42), it is interesting to notice that both IL-10 and glucocorticoids can polarize macrophages into an immunoregulatory profile (43). Based on that, we propose that the establishment of a foamy phenotype is accompanied by the acquisition of immunosuppressive properties.

It should be mentioned that Stattic is not an inhibitor completely specific for STAT3 (44). In this regard, a desirable goal is the discrimination between STAT3 and STAT1 involvement because both transcription factors can be activated by IL-10 (albeit at different levels), and because of their high degree of homology, particularly in their SH2 domains (44). Under our experimental conditions, unlike the strong activation of STAT3, we did not observe the phosphorylation of STAT1 in macrophages treated with TB-PE (data not shown and Figure 6). Likewise, the depletion of IFN-γ (a STAT1 activating cytokine) from TB-PE did not prevent the accumulation lipid bodies in macrophages (Figures 3A–C). Therefore, we consider that our experimental model is dependent of STAT3 instead of STAT1.

Our findings are in agreement with previous reports that demonstrated that macrophages exposed to lipids displayed impaired immune functions. Particularly, FM generated *in vitro* by the incubation with acetylated LDL displayed a reduced expression of pro-inflammatory genes (45). In addition, the ligation of the pregnane X receptor in human macrophages, which was associated to foamy formation, resulted in the impairment of the secretion of pro-inflammatory cytokines, phagolysosomal fusion and apoptosis (46). Moreover, a recent study showed that the treatment of human macrophages with surfactant lipids resulted in the reduction of TNF-α release and the enhancement of Mtb growth (14). Likewise, cholesterol-exposed THP1 macrophages failed both to produce TNF-α in response to Mtb and to clear the infection (15). To the best of our knowledge, we provide the first evidence that FM display a reduced ability to activate a recall Th1 response of specific antimycobacterial T cell clones. Therefore, we propose that FM significantly subvert the host immune response by impairing both the innate and the adaptive immune branches, and we predict a close relationship between lipid exposure, foamy phenotype acquisition and immunosuppressive properties.

Our study also provides additional mechanisms by which the environment created during infection can drive the foamy differentiation even in the absence of a direct contact with the pathogen. On the one hand, Mtb-infected macrophages can acquire a foamy phenotype as demonstrated in this study and several others (6–9, 27). Indeed, the accumulation of lipid bodies within infected cells has undesirable effects for the host, such as the protection of the pathogen against microbicidal mechanisms (10) and the acquisition of a dormancy phenotype, which confers tolerance to several front-line antibiotics (9). On the other hand, uninfected macrophages can also be driven into foamy cells by IL-10 and a source of lipids. These uninfected lipid-rich cells abrogate the host innate and adaptive cellular defense mechanisms, and when infected, they become a niche favoring pathogen persistence. In the latter case, we infer that uninfected individuals suffering from a lipid dysbalance may bear an enlarged pool of lipid-rich cells that potentially increase the susceptibility, persistence and/or progression of TB. In fact, diabetes and obesity have been associated with TB disease progression (47, 48), and even asymptomatic dyslipidemia was correlated to a reduced antimycobacterial activity (15).

In the past, most reports focused on assessing the impact of certain cytokines in the accumulation of lipid bodies in macrophages within the context of atherosclerosis; FM were proposed to cause the formation of atheroma (49). In fact, IL-1β and TNF-α are known to impede neutral lipid turnover in THP-1 cells loaded with lipoproteins (50), and these pro-inflammatory cytokines can decrease the efflux of lipids in J774 murine macrophages (51). In addition, IL-10 was shown to regulate lipid metabolism in human macrophages loaded with acetylated and oxidized LDL by increasing both cholesterol uptake and efflux resulting in a net increase in cholesterol content (52, 53). Considering that both pro- and anti-inflammatory cytokines were associated to the accumulation of lipid bodies, we evaluated in this study the effect of depleting several cytokines from TB-PE on the FM formation. In our hands, unlike the depletion of IL-10, the foamy phenotype induced by TB-PE was not altered by depletion of IL-1β, TNF-α, IL-6 or IFN-γ, pointing toward a specific role of IL-10 in promoting FM formation in the context of the pleural infection. Moreover, anti-inflammatory cytokines such as IL-4 and IL-13 were shown to alter lipid metabolism in macrophages through the activation of the lipid-activated nuclear receptors PPARγ (54), which mediates accumulation of lipid bodies (55, 56). Yet, we dismiss a potential role for IL-4 in our model given that its level was undetectable in TB-PE samples (data not shown).

In summary, our present study provides insights into the mechanisms by which host factors can enhance FM formation in macrophages. This knowledge may contribute to the identification of host molecular pathways that could be modulated to the benefit of the patient. Besides, the complementation of the conventional anti-TB therapy with host-directed therapies is desirable in order to achieve shorter treatment times, reduction in lung damage caused by the disease, and lower risk of relapse or reinfection. In this regard, a better understanding of the molecular mechanisms underlying host–pathogen interactions could provide a rational basis for the development of effective anti-TB therapeutics.

## Ethics Statement

Human samples: The research was carried out in accordance with the Declaration of Helsinki (2013) of the World Medical Association, and was approved by the Ethics Committees of the Hospital F. J Muñiz and the Academia Nacional de Medicina de Buenos Aires (protocol number: NIN-1671-12). Written informed consent was obtained before sample collection. Mice samples: Animals were bred and housed in accordance with the guidelines established by the Institutional Animal Care and Use Committee of Institute of Experimental Medicine (IMEX)-CONICET-ANM. All animal procedures were shaped to the principles set forth in the Guide for the Care and Use of Laboratory Animals (NIH, 1996).

## Author Contributions

MG designed experiments; MG, JF, MD, DK, AM, DM-E, EG-D, and CC performed experiments and analyzed data; BR, AF, and MM performed experiments with mice; EM, SP, and DP collected and provided TB samples; AA and GG performed TLC determinations; JC and RH-P performed ME determinations; PS, PB, ON, IM-P, CS-T, CC, and RH-P supervised experiments and wrote sections of the manuscript; CV, GL-V, MS, and LB contributed to conception and design of the study, supervised experiments, and wrote the manuscript. All authors contributed to manuscript revision, read, and approved the submitted version.

## Conflict of Interest Statement

Authors declare that the submitted work was carried out in the absence of personal, professional, or financial relationships that could potentially be construed as a conflict of interest.

## Supplementary Material

FIGURE S1 Interleukin-10 (IL-10) promotes lipid bodies’ accumulation in TB-PE-treated macrophages. **(A)** TB-PE were incubated with neutralizing antibodies for 1 h (4°C) for the depletion of IL-10, IL-6, IL-1β, or TNF-α, and then, Protein G Sepharose beads were added and incubated for 1 h (4°C). Finally, TB-PE was centrifuged at 12,000 × *g* to remove antibody-bead complexes. In the case of IFN-γ depletion, it was performed by incubating TB-PE for 2 h in sterile 96-well plates that had been coated with the capture antibody provided by the human IFN-γ ELISA Kit. In all cases, depletions were controlled by ELISA. **(B)** Human monocyte-derived macrophages were treated with TB-PE depleted or not of IL-10 for 24 h and then, cells were labeled with BODIPY 493/503 to visualize the lipid bodies by green fluorescence emission. The left panels are DIC images of the same field.

FIGURE S2 Correlation between cholesterol levels and different cytokines present in TB-PE. Correlation analysis between the levels of IL-6, IL-1β, TNF-α, or IFN-γ and the cholesterol content found in individual preparations of TB-PE (*n* = 23–24).

Figure S3 Signal transducer and activator of transcription 3 (STAT3) activation enhances lipid bodies accumulation by TB-PE. **(A)** Immunoblot images of p705-STAT3, STAT3, and β-actin (left panel); quantification of p705-STAT3 vs STAT3 on macrophages treated or not with Static (20 µM) or cucurbitacin (100 nM) for 2 h and then exposed or not to TB-PE for 24 h (right panel; *n* = 3). **(B)** Macrophages were treated or not with different concentrations of cucurbitacin for 2 h and then were exposed or not to TB-PE for 24 h. Lipid bodies’ content was assessed by Oil Red O (ORO) staining. The results are shown like the integrated density of ORO staining (*n* = 6) (**p* ≤ 0.05).
