# Supplementary figures and images for: Formation of Foamy Macrophages by Tuberculous Pleural Effusions Is Triggered by the Interleukin-10/Signal Transducer and Activator of Transcription 3 Axis through ACAT Upregulation
Source: Front Immunol. 2018 Mar 9;9:459. doi: 10.3389/fimmu.2018.00459 (PMC5854656; doi:10.3389/fimmu.2018.00459)

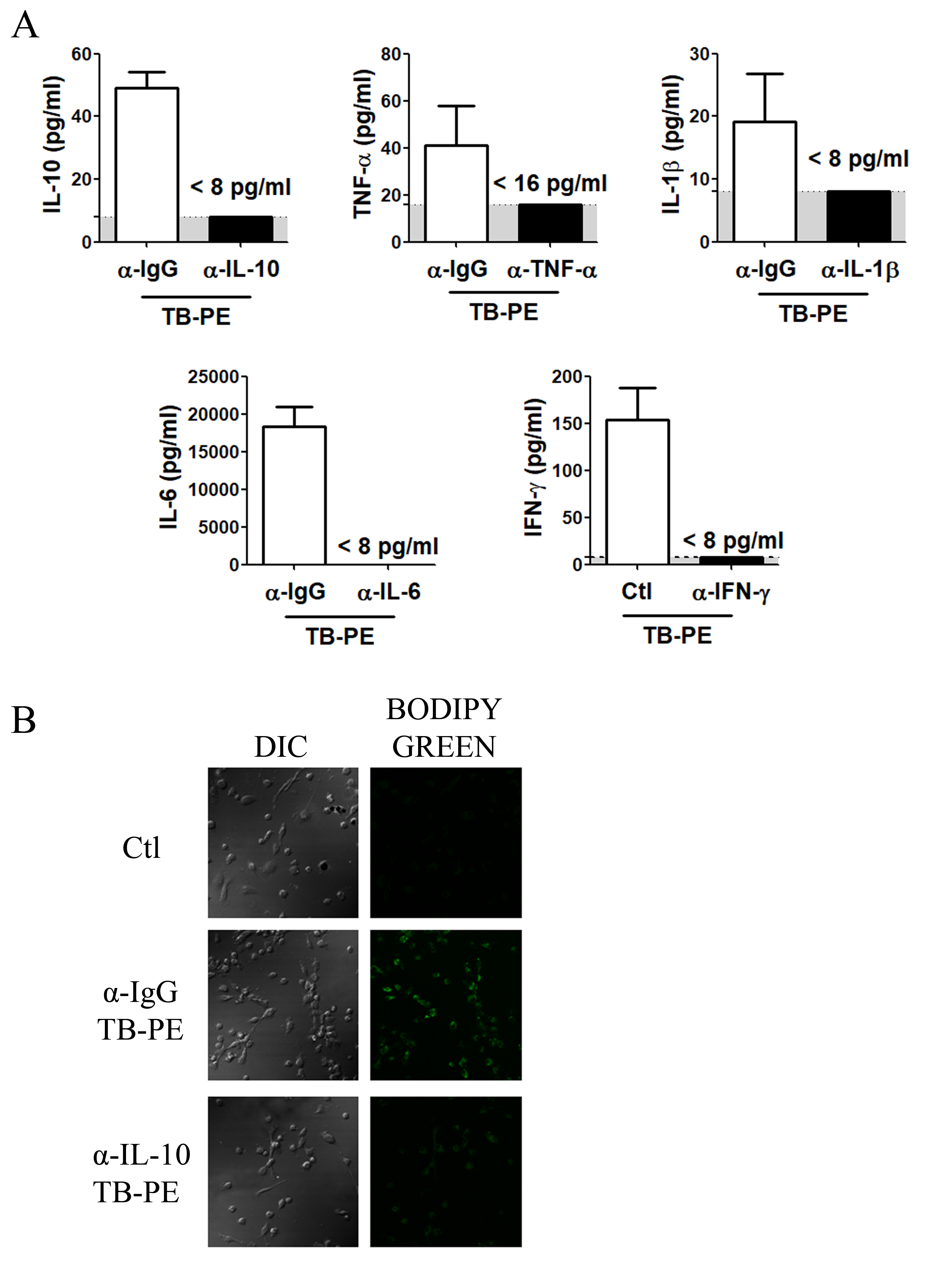

Supplement: FIGURE S1 — Interleukin-10 (IL-10) promotes lipid bodies’ accumulation in TB-PE-treated macrophages. (A) TB-PE were incubated with neutralizing antibodies for 1 h (4°C) for the depletion of IL-10, IL-6, IL-1β, or TNF-α, and then, Protein G Sepharose beads were added and incubated for 1 h (4°C). Finally, TB-PE was centrifuged at 12,000 × g to remove antibody-bead complexes. In the case of IFN-γ depletion, it was performed by incubating TB-PE for 2 h in sterile 96-well plates that had been coated with the capture antibody provided by the human IFN-γ ELISA Kit. In all cases, depletions were controlled by ELISA. (B) Human monocyte-derived macrophages were treated with TB-PE depleted or not of IL-10 for 24 h and then, cells were labeled with BODIPY 493/503 to visualize the lipid bodies by green fluorescence emission. The left panels are DIC images of the same field. [file image_1.tif]

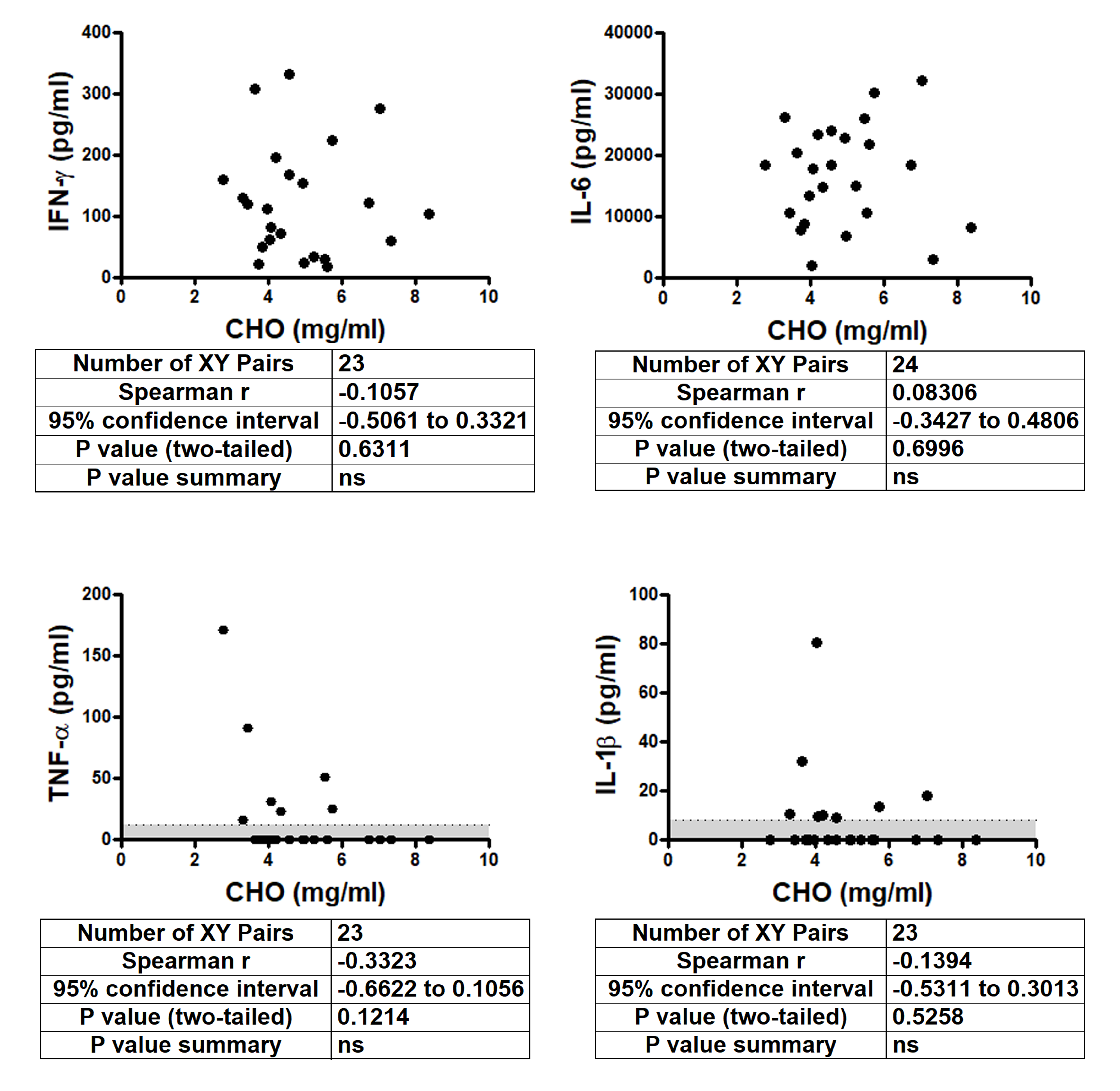

Supplement: FIGURE S2 — Correlation between cholesterol levels and different cytokines present in TB-PE. Correlation analysis between the levels of IL-6, IL-1β, TNF-α, or IFN-γ and the cholesterol content found in individual preparations of TB-PE (n = 23–24). [file image_2.tif]

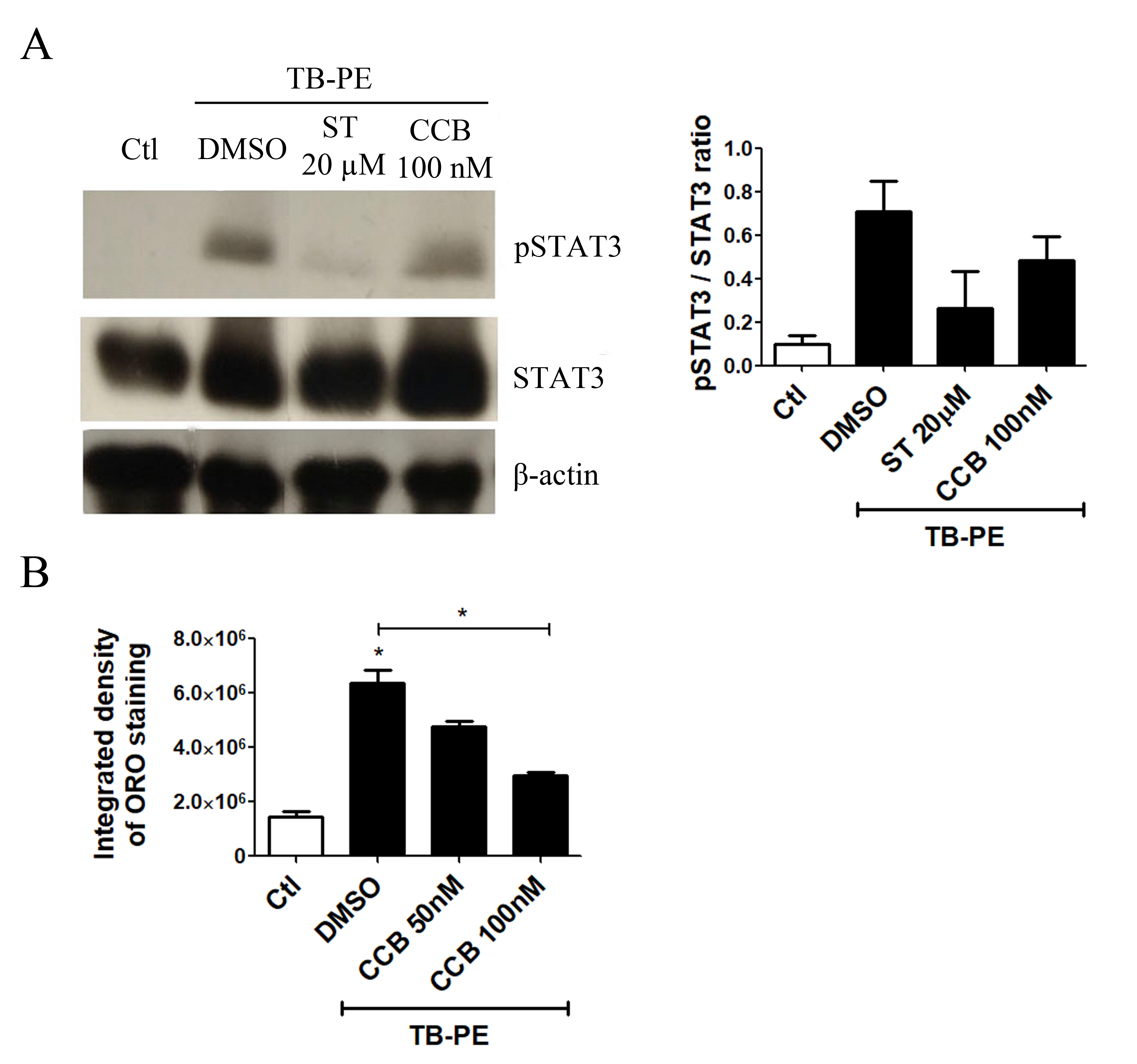

Supplement: Figure S3 — Signal transducer and activator of transcription 3 (STAT3) activation enhances lipid bodies accumulation by TB-PE. (A) Immunoblot images of p705-STAT3, STAT3, and β-actin (left panel); quantification of p705-STAT3 vs STAT3 on macrophages treated or not with Static (20 µM) or cucurbitacin (100 nM) for 2 h and then exposed or not to TB-PE for 24 h (right panel; n = 3). (B) Macrophages were treated or not with different concentrations of cucurbitacin for 2 h and then were exposed or not to TB-PE for 24 h. Lipid bodies’ content was assessed by Oil Red O (ORO) staining. The results are shown like the integrated density of ORO staining (n = 6) (*p ≤ 0.05). [file image_3.tif]
